# Effect of Positive End-Expiratory Pressure on the Sonographic Optic Nerve Sheath Diameter as a Surrogate for Intracranial Pressure during Robot-Assisted Laparoscopic Prostatectomy: A Randomized Controlled Trial

**DOI:** 10.1371/journal.pone.0170369

**Published:** 2017-01-20

**Authors:** Ji-Hyun Chin, Wook-Jong Kim, Joonho Lee, Yun A. Han, Jinwook Lim, Jai-Hyun Hwang, Seong-Sik Cho, Young-Kug Kim

**Affiliations:** 1 Department of Anesthesiology and Pain Medicine, Asan Medical Center, University of Ulsan College of Medicine, Seoul, Republic of Korea; 2 Department of Occupational and Environmental Medicine, Konkuk University Chungju Hospital, Chungju, Republic of Korea; Ohio State University, UNITED STATES

## Abstract

**Background:**

Positive end-expiratory pressure (PEEP) can increase intracranial pressure. Pneumoperitoneum and the Trendelenburg position are associated with an increased intracranial pressure. We investigated whether PEEP ventilation could additionally influence the sonographic optic nerve sheath diameter as a surrogate for intracranial pressure during pneumoperitoneum combined with the Trendelenburg position in patients undergoing robot-assisted laparoscopic prostatectomy.

**Methods:**

After anesthetic induction, 38 patients were randomly allocated to a low tidal volume ventilation (8 ml/kg) without PEEP group (zero end-expiratory pressure [ZEEP] group, n = 19) or low tidal volume ventilation with 8 cmH_2_O PEEP group (PEEP group, n = 19). The sonographic optic nerve sheath diameter was measured prior to skin incision, 5 min and 30 min after pneumoperitoneum and the Trendelenburg position, and at the end of surgery. The study endpoint was the difference in the sonographic optic nerve sheath diameter 5 min after pneumoperitoneum and the Trendelenburg position between the ZEEP and PEEP groups.

**Results:**

Optic nerve sheath diameters 5 min after pneumoperitoneum and the Trendelenburg position did not significantly differ between the groups [least square mean (95% confidence interval); 4.8 (4.6–4.9) mm vs 4.8 (4.7–5.0) mm, P = 0.618]. Optic nerve sheath diameters 30 min after pneumoperitoneum and the Trendelenburg position also did not differ between the groups [least square mean (95% confidence interval); 4.5 (4.3–4.6) mm vs 4.5 (4.4–4.6) mm, P = 0.733].

**Conclusions:**

An 8 cmH_2_O PEEP application under low tidal volume ventilation does not induce an increase in the optic nerve sheath diameter during pneumoperitoneum combined with the steep Trendelenburg position, suggesting that there might be no detrimental effects of PEEP on the intracranial pressure during robot-assisted laparoscopic prostatectomy.

**Trial Registration:**

ClinicalTrial.gov NCT02516566

## Introduction

Robot-assisted laparoscopic prostatectomy requires a pneumoperitoneum and the steep Trendelenburg position to facilitate a surgical field. These specific conditions induce decreased pulmonary functional residual capacity and pulmonary compliance [1], which are likely to impose postoperative respiratory complications. The lung protective ventilation strategy during surgery, which consists of low tidal volume and positive end-expiratory pressure (PEEP) ventilation, has been known to improve postoperative respiratory outcomes in diverse surgical patients, including those undergoing laparoscopic surgery, who are at high risk for postoperative respiratory complications [2,3].

Although the lung protective ventilation strategy during laparoscopic surgery is promising in its potential to improve outcomes in regards to pulmonary function, the application of PEEP as an important component of this strategy does not necessarily favor the function of other major organs. In particular, it has been proposed that PEEP results in increased intracranial pressure (ICP) by impeding cerebrospinal flow outflow and cerebral venous drainage [4]. Furthermore, recent studies have reported that the sonographic optic nerve sheath diameter (ONSD), which reflects ICP, increased during robot-assisted laparoscopic prostatectomy [5,6]. Thus, the application of PEEP may induce an additional increase in ICP during pneumoperitoneum and the steep Trendelenburg position in patients undergoing robot-assisted laparoscopic prostatectomy. However, no reports have evaluated the relationship between PEEP and ICP during specific conditions such as pneumoperitoneum and the steep Trendelenburg position.

In the present study, we sought to evaluate the effect of PEEP on the ONSD as a surrogate for ICP during pneumoperitoneum and the steep Trendelenburg position in patients who have undergone a robot-assisted laparoscopic prostatectomy.

## Materials and Methods

### Patients

This monocentric, parallel-group, randomized controlled trial was conducted between September and October 2015. The study protocol was approved by the Institutional Review Board at Asan Medical Center (2015–0741) and written informed consent was obtained from each patient. This study was also registered with ClinicalTrial.gov (NCT02516566). Patients scheduled for robot-assisted laparoscopic prostatectomy were enrolled and evaluated. Patients with cerebrovascular disease, glaucoma, or who refused to participate were excluded. Patients who were younger than 20 years were also excluded.

After applying routine hemodynamic monitoring (three-lead electrocardiogram, noninvasive blood pressure, and pulse oximetry) and attaching the cerebral oximeter sensors (INVOS 5100) to the right and left frontal areas, anesthesia was induced using a bolus intravenous injection of 5 mg/kg thiopental sodium followed by 0.6 mg/kg rocuronium. After tracheal intubation, patients were mechanically ventilated with a tidal volume of 8 ml/kg ideal body weight and a respiratory rate to maintain end-tidal CO_2_ partial pressure (ETCO_2_) between 30 and 40 mmHg. The inspiratory-to-expiratory time ratio was set at 1:2. Anesthesia was maintained with end-tidal sevoflurane concentration of 2 vol% plus the continuous infusion of remifentanil. Remifentanil was infused in the range of 0.07–0.16 μg/kg/min which was titrated to maintain hemodynamic stability. A 50% oxygen was supplied using medical air.

### Randomization

**Fig 1:**
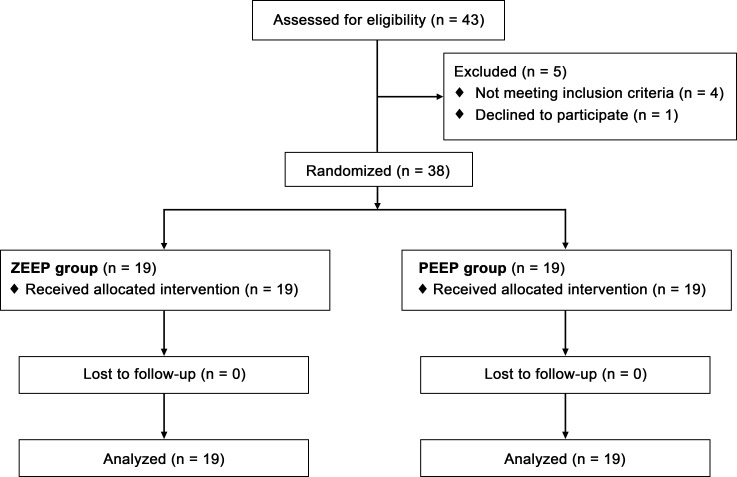
Study flow diagram. Patients in the zero end-expiratory pressure (ZEEP) group received mechanical ventilation with a tidal volume 8 ml/kg of ideal body weight without positive end-expiratory pressure (PEEP), and those in PEEP group received mechanical ventilation with a tidal volume 8 ml/kg of ideal body weight with 8 cmH_2_O PEEP.

One investigator (W-JK) generated a randomization code using block randomization procedure with 1:1 allocation ratio. Random allocation was carried out with the user-developed Stata module (RALLOC: Stata module to design randomized controlled trials). The participants were assigned to randomization codes kept in sequentially numbered opaque envelopes. After anesthetic induction, these envelops were opened by an investigator who controlled ventilator setting, and 38 patients were randomly allocated to one of two groups: the zero end-expiratory pressure (ZEEP) group (n = 19) which received mechanical ventilation with a tidal volume of 8 ml/kg of ideal body weight without PEEP, and the PEEP group (n = 19) which received mechanical ventilation with a tidal volume of 8 ml/kg of ideal body weight with 8 cmH_2_O PEEP (Fig 1). One investigator controlled the ventilator setting according to a randomization code and other investigators who were blinded to the ventilator setting, which was concealed by a screen, measured the ONSD.

### Ocular sonography

The ONSD was measured by investigators trained in ocular sonography. Briefly, patients were placed in the supine position with their eyes closed, and a thick gel layer was applied to the closed upper eyelid. A 7.5 MHz linear probe was placed on the gel without excessive pressure and adjusted to the proper angle to display the optimal contrast between the retrobulbar echogenic fat tissue and the vertical hypoechoic band. An ultrasound beam was focused onto the retrobulbar area using the lowest possible acoustic power that could measure the ONSD. The ONSD was measured 3 mm behind the optic disc. Measurements were performed in the transverse and sagittal planes of both eyes, and the mean values of four measurements at each time point were used in the analysis. To determine intra-observer and inter-observer variability, a random sample of about 25% of the ONSD was submitted twice to the first investigator and once to a second investigator. The inter-observer variability was then calculated as the mean absolute difference between the two readings from the first and second investigator divided by their mean and expressed as a percentage. Similarly, the intra-observer variability was calculated as the mean absolute difference between the two readings from the first investigator divided by their mean and expressed as a percentage.

### Study protocol

When hemodynamically stable conditions were reached, measurements were taken as follows: 10 min after anesthetic induction in the supine position before random allocation (T0), 5 min after applying ventilation strategies according to a random allocation (T1), 5 min after establishing pneumoperitoneum (15 mmHg of insufflation pressure) and the steep Trendelenburg position (35° incline) (T2), 30 min after establishing pneumoperitoneum and the steep Trendelenburg position (T3), and at the end of surgery after the desufflation of pneumoperitoneum in the supine position (T4). At each predetermined time point, we measured the following variables; ONSD, mean arterial blood pressure, heart rate, tympanic body temperature, static pulmonary compliance (tidal volume/[airway plateau pressure-PEEP]), dynamic pulmonary compliance (tidal volume/[airway peak pressure-PEEP]), ETCO_2_, arterial CO_2_ partial pressure, arterial O_2_ partial pressure (PaO_2_), hemoglobin concentration, and regional cerebral oxygen saturation (rSO_2_) using near infrared spectroscopy.

### Study endpoint

The study endpoint was the difference in ONSD measurements at T2 (5 min after establishing pneumoperitoneum and the Trendelenburg position) between the ZEEP and PEEP groups.

### Statistical analysis

Our previous study reported an ONSD during pneumoperitoneum and the Trendelenburg position of 4.9 mm (SD: 0.4 mm) [5]. We assumed a 10% increase in the ONSD at T2 in the PEEP group compared with that in the ZEEP group, with reference to a previous study of patients who received a 7–8 ml/kg tidal volume with 8 cmH_2_O PEEP [7]. A 10% increase in the ONSD is a level which could reflect an increased ICP (> 20 mmHg) [8,9]. Power analysis suggested that a minimum sample size of 34 patients would be required to detect a 0.5 mm (about 10% of 4.9 mm) difference in the mean ONSD between the ZEEP and PEEP groups with a power of 80% at a P < 0.05 level of significance. Expecting a dropout rate of about 10%, we aimed to include 38 patients. The linear mixed effect model was used to compare changes in ONSD, hemodynamic variables, respiratory variables, and rSO_2_ within and between the groups. The data regarding ONSD, hemodynamic variables, respiratory variables, and rSO_2_ were presented as a least square mean (95% confidence interval). A P value < 0.05 was considered statistically significant. All of the statistical analyses were performed using SigmaPlot software, version 12.5 (Systat Software Inc, San Jose, CA) and Stata software version 13.1 (StataCorp LP, College Station, TX).

## Results

During the study period, 43 patients were enrolled. Of these, 1 declined to participate and 4 were excluded due to cerebrovascular disease. A final total of 38 patients was therefore randomly allocated to the two groups and completed the study protocol (Fig 1). There were no significant demographic differences between the two groups, as indicated in Table 1.

**Table 1:** Demographic data.

	ZEEP group (n = 19)	PEEP group (n = 19)
Age (yr)	65.1 ± 6.9	63.0 ± 6.8
Weight (kg)	70.3 ± 9.4	73.4 ± 10.2
Height (cm)	165.4 ± 7.4	169.8 ± 4.8
Body mass index (kg/m^2^)	25.7 ± 3.1	25.5 ± 3.5

Data are expressed as mean ± SD. ZEEP group = zero end-expiratory pressure with low tidal volume ventilation; PEEP group = positive end-expiratory pressure with low tidal volume ventilation.

At baseline (T0), the ONSDs were not significantly different between the ZEEP and PEEP groups (P = 0.245) (Table 2). At 5 min after applying the allocated ventilation strategies in the supine position without pneumoperitoneum (T1), the ONSD in the PEEP group increased compared to that in the ZEEP group [4.4 (4.2–4.5) mm vs 4.1 (4.0–4.3) mm, P = 0.021] (Table 2).

**Table 2:** Sonographic optic nerve sheath diameter in the ZEEP and PEEP groups during robot-assisted laparoscopic prostatectomy.

	ZEEP group	PEEP group	Estimated Difference (95% CI)	P
T0	4.1 (3.9–4.2)	4.2 (4.1–4.3)	0.1 (-0.1–0.3)	0.245
T1	4.1 (4.0–4.3)	4.4 (4.2–4.5)	0.2 (0.0–0.4)	0.021
T2	4.8 (4.6–4.9)	4.8 (4.7–5.0)	0.1 (-0.1–0.2)	0.618
T3	4.5 (4.3–4.6)	4.5 (4.4–4.6)	0.0 (-0.2–0.2)	0.733
T4	4.3 (4.2–4.5)	4.4 (4.3–4.5)	0.1 (-0.1–0.2)	0.599

Values are the least square mean (95% confidence interval). CI = confidence interval; ZEEP group = zero end-expiratory pressure with low tidal volume ventilation; PEEP group = positive end-expiratory pressure with low tidal volume ventilation; T0 = 10 minutes after anesthetic induction in the supine position before random allocation; T1 = 5 minutes after applying ventilation strategies according to a random allocation; T2 = 5 minutes after establishing pneumoperitoneum and the steep Trendelenburg position; T3 = 30 minutes after establishing pneumoperitoneum and the steep Trendelenburg position; T4 = at the end of surgery after desufflation of pneumoperitoneum in the supine position.

**Fig 2:**
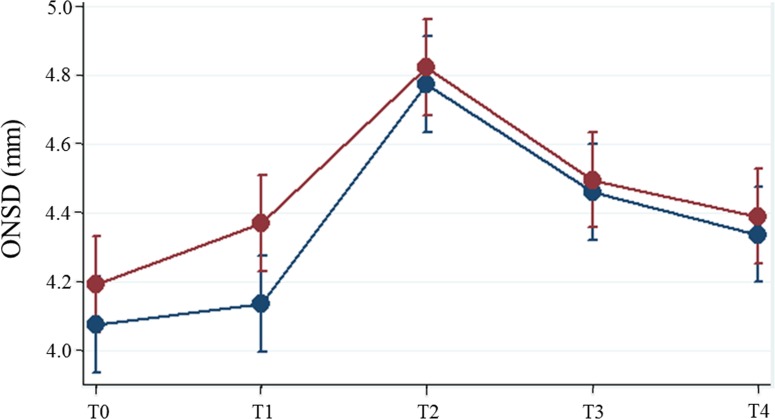
Changes in ONSD in the ZEEP group (blue circle) and PEEP group (red circle) during robot-assisted laparoscopic prostatectomy. Note that the ONSDs during pneumoperitoneum and the Trendelenburg position (T2 and T3) do not significantly differ between the ZEEP and PEEP groups, whereas the ONSD in the PEEP group is significantly higher than that in the ZEEP group during the supine position without pneumoperitoneum (T1). Circles and bars indicate least square means and 95% confidence intervals, respectively. ONSD = optic nerve sheath diameter; ZEEP group = zero end-expiratory pressure with low tidal volume ventilation; PEEP group = positive end-expiratory pressure with low tidal volume ventilation; T0 = 10 min after anesthetic induction in the supine position before random allocation; T1 = 5 min after applying ventilation strategies according to a random allocation; T2 = 5 min after establishing pneumoperitoneum and the steep Trendelenburg position; T3 = 30 min after establishing pneumoperitoneum and the steep Trendelenburg position; T4 = at the end of surgery after desufflation of pneumoperitoneum in the supine position.

The ONSDs during pneumoperitoneum and the steep Trendelenburg position (T2 and T3) showed no significant differences between the ZEEP and PEEP groups [at T2, 4.8 (4.6–4.9) mm vs 4.8 (4.7–5.0) mm, P = 0.618; at T3, 4.5 (4.3–4.6) mm vs 4.5 (4.4–4.6) mm, P = 0.733] (Fig 2). Similarly, the ONSDs at the end of surgery (T4) showed no significant difference between PEEP and ZEEP groups (P = 0.599) (Table 2). The intra- and inter-observer variabilities of measuring the ONSD were 2.2% and 3.7%, respectively.

At 5 min after applying ventilation strategies during the supine position without pneumoperitoneum (T1), the PaO_2_ level in the PEEP group tended to increase compared with that in ZEEP group (P = 0.104) (Table 3). At T1, both static and dynamic pulmonary compliance in the PEEP group were significantly higher than those in ZEEP group (both P < 0.001). During pneumoperitoneum and the steep Trendelenburg position (T2 and T3), the PaO_2_ level and static and dynamic pulmonary compliance in the PEEP group tended to increase compared with these measurements in the ZEEP group (Table 3).

**Table 3:** Variables in the ZEEP and PEEP groups during robot-assisted laparoscopic prostatectomy.

	ZEEP group	PEEP group	Estimated difference (95% CI)	P
MBP (mmHg)				
T0	74.1 (69.8–78.4)	76.3 (71.9–80.6)	2.2 (-4.0–8.3)	0.490
T1	70.0 (65.6–74.3)	71.5 (67.1–75.8)	1.5 (-4.7–7.6)	0.637
T2	91.8 (87.5–96.1)	87.8 (83.5–92.2)	-3.9 (-10.1–2.2)	0.207
T3	77.3 (73.0–81.7)	79.7 (75.4–84.1)	2.4 (-3.7–8.5)	0.442
T4	73.2 (68.9–77.5)	76.5 (72.1–80.8)	3.2 (-2.9–9.4)	0.299
HR (beats/min)				
T0	68.1 (64.0–72.2)	69.3 (65.2–73.3)	1.2 (-4.6–6.9)	0.692
T1	63.0 (58.9–67.1)	64.6 (60.5–68.6)	1.6 (-4.2–7.3)	0.590
T2	57.2 (53.2–61.3)	59.5 (55.5–63.6)	2.3 (-3.4–8.1)	0.429
T3	58.0 (53.9–62.1)	59.3 (55.2–63.3)	1.3 (-4.5–7.0)	0.666
T4	62.7 (58.6–66.7)	61.8 (57.8–65.9)	-0.8 (-6.6–4.9)	0.774
BT (°C)				
T0	36.0 (35.8–36.2)	36.0 (35.8–36.2)	0.0 (-0.3–0.3)	1.000
T1	36.1 (35.9–36.3)	36.1 (35.9–36.3)	0.0 (-0.3–0.3)	1.000
T2	36.1 (35.9–36.3)	36.1 (36.0–36.3)	0.03 (-0.2–0.3)	0.842
T3	36.1 (35.9–36.2)	36.1 (35.9–36.3)	0.1 (-0.2–0.3)	0.690
T4	36.0 (35.8–36.1)	36.1 (35.9–36.3)	0.1 (-0.1–0.4)	0.360
ETCO_2_ (mmHg)				
T0	30.7 (30.0–31.5)	30.5 (29.8–31.3)	-0.2 (-1.3–0.9)	0.703
T1	30.6 (29.8–31.3)	30.5 (29.8–31.3)	-0.1 (-1.1–1.0)	0.924
T2	31.7 (30.9–32.5)	31.7 (30.9–32.5)	0.0 (-1.1–1.1)	1.000
T3	31.5 (30.7–32.2)	31.3 (30.5–32.1)	-0.2 (-1.2–0.9)	0.775
T4	32.6 (31.9–33.4)	33.1 (32.3–33.8)	0.4 (-0.7–1.5)	0.447
PaCO_2_ (mmHg)				
T0	40.8 (39.7–42.0)	40.3 (39.1–41.5)	-0.5 (-2.2–1.1)	0.537
T1	39.7 (38.5–40.9)	37.5 (36.3–38.7)	-2.2 (-3.8–-0.5)	0.011
T2	42.1 (40.9–43.2)	41.8 (40.6–43.0)	-0.3 (-1.9–1.4)	0.757
T3	42.1 (40.9–43.3)	41.1 (39.9–42.3)	-1.0 (-2.7–0.7)	0.241
T4	43.6 (42.5–44.8)	42.8 (41.6–44.0)	-0.8 (-2.5–0.8)	0.323
PaO_2_ (mmHg)				
T0	225.7 (201.1–250.4)	239.6 (213.9–265.4)	13.9 (-21.7–49.5)	0.444
T1	191.9 (167.3–216.6)	221.1 (196.0–246.2)	29.2 (-6.0–64.3)	0.104
T2	156.5 (131.9–181.1)	190.8 (166.2–215.5)	34.4 (-0.4–69.2)	0.053
T3	152.3 (127.7–176.9)	181.3 (156.7–205.9)	29.0 (-5.8–63.8)	0.103
T4	155.7 (131.1–180.3)	192.1 (167.5–216.7)	36.4 (1.6–71.2)	0.040
Hgb (g/dl)				
T0	13.0 (12.4–13.7)	13.3 (12.7–14.0)	0.3 (-0.6–1.2)	0.520
T1	12.7 (12.1–13.4)	12.7 (12.1–13.4)	0.0 (-0.9–0.9)	1.000
T2	12.9 (12.3–13.6)	13.1 (12.4–13.8)	0.2 (-0.8–1.1)	0.721
T3	12.5 (11.8–13.1)	12.8 (12.2–13.5)	0.4 (-0.6–1.3)	0.441
T4	12.0 (11.3–12.7)	12.3 (11.6–12.9)	0.3 (-0.7–1.2)	0.584
rSO_2_ (%)				
T0	70.7 (67.7–73.6)	73.1 (70.1–76.0)	2.4 (-1.8–6.6)	0.265
T1	67.8 (64.8–70.7)	68.2 (65.3–71.2)	0.5 (-3.7–4.7)	0.825
T2	66.9 (63.9–69.9)	68.6 (65.6–71.5)	1.6 (-2.6–5.8)	0.447
T3	66.5 (63.5–69.5)	67.8 (64.8–70.8)	1.3 (-2.9–5.5)	0.540
T4	65.9 (63.0–68.9)	68.7 (65.7–71.7)	2.8 (-1.4–7.0)	0.198
C_stat_ (ml/cmH_2_O)				
T0	42.2 (39.5–47.0)	44.0 (40.3–47.8)	0.8 (-4.5–6.1)	0.766
T1	43.4 (39.7–47.2)	55.6 (51.9–59.4)	12.2 (6.9–17.5)	<0.001
T2	18.2 (14.5–22.0)	23.4 (19.6–27.1)	5.2 (-0.1–10.5)	0.057
T3	18.3 (14.6–22.1)	23.7 (20.0–27.5)	5.4 (0.1–10.7)	0.046
T4	33.1 (29.4–36.9)	44.6 (40.8–48.3)	11.5 (6.2–16.8)	<0.001
C_dyn_ (ml/cmH_2_O)				
T0	42.1 (38.6–45.5)	42.5 (39.0–46.0)	0.4 (-4.5–5.3)	0.867
T1	42.1 (38.6–45.6)	54.6 (51.1–58.0)	12.4 (7.5–17.4)	<0.001
T2	17.9 (14.4–21.4)	23.0 (19.5–26.5)	5.1 (0.2–10.0)	0.042
T3	17.9 (14.5–21.4)	22.4 (18.9–25.9)	4.4 (-0.5–9.4)	0.076
T4	31.0 (27.5–34.4)	39.9 (36.4–43.4)	8.9 (4.0–13.9)	<0.001

Values are the least square mean (95% confidence interval). CI = confidence interval; ZEEP group = zero end-expiratory pressure with low tidal volume ventilation; PEEP group = positive end-expiratory pressure with low tidal volume ventilation; MBP = mean arterial blood pressure; HR = heart rate; BT = body temperature; ETCO_2_ = end-tidal CO_2_ partial pressure; PaCO_2_ = arterial CO_2_ partial pressure; PaO_2_ = arterial O_2_ partial pressure; Hgb = hemoglobin concentration; rSO_2_ = regional cerebral oxygen saturation; C_stat_ = static pulmonary compliance; C_dyn_ = dynamic pulmonary compliance; T0 = 10 minutes after anesthetic induction in the supine position before random allocation; T1 = 5 minutes after applying ventilation strategies according to a random allocation; T2 = 5 minutes after establishing pneumoperitoneum and the steep Trendelenburg position; T3 = 30 minutes after establishing pneumoperitoneum and the steep Trendelenburg position; T4 = at the end of surgery after desufflation of pneumoperitoneum in the supine position.

## Discussion

We found that an 8 cmH_2_O PEEP application under low tidal volume ventilation did not induce an increase in the ONSD during the specific conditions of pneumoperitoneum and the steep Trendelenburg position. In addition, the 8 cmH_2_O PEEP application resulted in an increase in the ONSD in the supine position without pneumoperitoneum.

PEEP is a lung protective ventilation strategy component that has been known to reduce postoperative respiratory complications in patients who are at high risk for adverse events during abdominal surgeries including laparoscopic surgery [2,3]. PEEP applied to the airway might be transmitted to the intrathoracic blood, resulting in higher intrathoracic pressure and central venous pressure, and subsequent increases in ICP [4]. Therefore, the use of PEEP has generated concerns regarding ICP changes. Previous studies have investigated the effects of PEEP on ICP in several clinical settings including high ICP patients with a head injury as well as normal ICP patients. It has been reported that a PEEP of up to 12 cmH_2_O did not significantly influence ICP in patients with acute stroke [10]. In another study of subarachnoid hemorrhage patients without vasospasm, a PEEP of up to 20 cmH_2_O had no significant impact on ICP [11]. Interestingly, a further study reported that a PEEP of up to 15 cmH_2_O did not increase the ICP in patients with a high ICP, whereas the same levels of PEEP increased the ICP in patients with a normal ICP [4].

Robot-assisted laparoscopic prostatectomy requires a pneumoperitoneum and the steep Trendelenburg position. Although these specific conditions are necessary for a good surgical field, they can induce pathophysiologic changes in major organs including the central nervous system [12]. However, studies on the effects of these specific conditions on the sonographic ONSD, as a noninvasive and simple surrogate for ICP, have shown conflicting results. A previous study reported that the ONSD did not increase during pneumoperitoneum and the Trendelenburg position [13]. In contrast, it has recently been reported that the sonographic ONSD increased in patients undergoing laparoscopic prostatectomy under pneumoperitoneum and the steep Trendelenburg position [5,6,14]. The increased intrathoracic pressure by a pneumoperitoneum and the Trendelenburg position might interfere with the cerebral venous drainage, resulting in an increased cerebral venous pressure and finally a higher ICP [15,16]. An increased ICP, resulted from cerebral venous congestion, does not necessarily lead to cerebral parenchymal edema in the case of patients with an intact blood-brain barrier. The patients without cerebral pathology might not be at risk for substantial cerebral complications, even with an ICP increase during pneumoperitoneum and the Trendelenburg position as was reported in some previous studies.

Our current study was initiated from a concern about additional increases in the ICP following a PEEP application during pneumoperitoneum combined with the steep Trendelenburg position. Our findings have indicated however that an 8 cmH_2_O PEEP under low tidal volume ventilation did not induce an additional increase in ONSD which already increased during pneumoperitoneum and the Trendelenburg position, implying no further detrimental effects of PEEP on an increased ICP during robot-assisted laparoscopic prostatectomy. A PEEP application might not increase the perioperative risks for cerebral complications in patients without cerebral pathology undergoing laparoscopic surgery. Additionally, we found that an 8 cmH_2_O PEEP resulted in an increase in ONSD in the supine position without pneumoperitoneum. Our present results are in line with those of a previous study that reported an increased ICP level in patients with a normal ICP following a PEEP application, whereas the same level of PEEP did not alter the ICP in patients with a high ICP [4]. Our findings might be explained by the waterfall model, in which ICP and intrathoracic pressure act as the upstream and downstream pressure, respectively [17]. In the supine position, an increased intrathoracic pressure by PEEP might exceed the ICP in our patients with a normal ICP. In contrast, during pneumoperitoneum and the steep Trendelenburg position, an increased intrathoracic pressure following PEEP might not reach to a level above an increased ICP, although PEEP increases the intrathoracic pressure per se. In addition, a decreased lung compliance observed in our patients might, at least in part, contribute to our finding of no significant effect of PEEP on ONSD during pneumoperitoneum and the steep Trendelenburg position. It has been reported in this regard that a transmission of PEEP to the intracranial component was attenuated in patients with decreased lung compliance [18]. Our current study patients showed a decreased lung compliance which might be caused by pneumoperitoneum, the steep Trendelenburg position, and a tightened chest wall due to use of straps.

We further found that ONSD at 30 minutes after initiating pneumoperitoneum combined with the steep Trendelenburg position had not increase further compared with that at 5 minutes after this position change; rather it was less than ONSD at 5 minutes after this position change. This observation might suggest that cerebral blood flow was probably adjusted over time during surgery, and partially compensated by cerebrospinal fluid translocation [11]. Maintenance of the ETCO_2_ at 30–40 mmHg by controlling the respiratory rate may have also contributed to the result observed 30 minutes after the patients were placed in this position. Since leaving ETCO_2_ as it had been during laparoscopic surgery without any deliberate manipulation could raise ethical issues, it was adjusted during surgery.

Our study had the following limitations. First, we did not measure the ICP directly, as this is not possible in non-neurosurgical patients due to ethical issues. Instead, we measured the sonographic ONSD that has been known to correlate with the directly measured ICP using ventriculostomy [19]. Second, we selected one level of PEEP (i.e. 8 cmH_2_O), which has been suggested previously by Futier et al. as part of a lung protective ventilation strategy [2]. It is possible that higher levels of PEEP induce an increase in intrathoracic pressure that exceeds an increased ICP during robot-assisted laparoscopic surgery. Therefore, further studies will be needed to evaluate the effect of various levels of PEEP on ICP during robot-assisted laparoscopic prostatectomy. Third, there was a possible selection bias in our study due to a lack of an allocation concealment. We did not implement a more specific method to prevent foreknowledge of the study group allocation (eg. placing cardboard or aluminum foil inside the envelope), although we used sequentially numbered opaque envelopes and opened those after anesthetic induction to provide allocation concealment.

In conclusion, an 8 cmH_2_O PEEP application under low tidal volume ventilation does not induce an ONSD increase during pneumoperitoneum combined with the steep Trendelenburg position. Our results suggest that PEEP under low tidal volume ventilation might be used with no additional risk of an unwanted increase in ICP in patients without cerebral pathology who are undergoing robot-assisted laparoscopic prostatectomy.

## Supporting Information

S1 CONSORT Checklist (DOC)

S1 File Dataset. (XLSX)

S1 Protocol Study Protocol. (DOC)
