# Supplementary material for: Effect of Positive End-Expiratory Pressure on the Sonographic Optic Nerve Sheath Diameter as a Surrogate for Intracranial Pressure during Robot-Assisted Laparoscopic Prostatectomy: A Randomized Controlled Trial
Source: PLoS One. 2017 Jan 20;12(1):e0170369. doi: 10.1371/journal.pone.0170369 (PMC5249217; doi:10.1371/journal.pone.0170369)
Supplement: S1 Protocol — (DOC) [file pone.0170369.s003.doc]

**Study protocol**

**Title**

Effects of Positive End-Expiratory Pressure Ventilation on Optic Nerve Sheath Diameter in Patients Undergoing Robot-Assisted Laparoscopic Prostatectomy

**Corresponding author**

Young-Kug Kim, MD, PhD

Professor, Department of Anesthesiology and Pain Medicine

Asan Medical Center, University of Ulsan College of Medicine

Seoul 05505, Republic of Korea

Tel: 82-2-3010-5976, Fax: 82-2-3010-6790, Email: kyk@amc.seoul.kr

**Summary**

| **Title** | Effects of Positive End-Expiratory Pressure Ventilation on Optic Nerve Sheath Diameter in Patients Undergoing Robot-Assisted Laparoscopic Prostatectomy |
| --- | --- |
| **Purpose** | The purpose of this study is to evaluate the effect of positive end-expiratory pressure as a lung protective ventilation strategy on the sonographic optic nerve sheath diameter as a surrogate for intracranial pressure during pneumoperitoneum and the steep Trendelenburg position in patients who are scheduled to undergo robot-assisted laparoscopic prostatectomy. |
| **Principal investigator** | Young-Kug Kim, MD, PhD |
| **Institute** | Asan Medical Center, University of Ulsan College of Medicine |
| **Sponsor** | None |
| **Patients** | Patients who are scheduled to undergo robot-assisted laparoscopic prostatectomy |
| **Clinical trial of drug** | None |
| **Study design** | Randomized controlled trial |
| **Measurement protocol** | 1. Measurements time points   T0: 10 min after anesthetic induction in the supine position  before random allocation  T1: 5 min after applying ventilation strategies according to a  random allocation  T2: 5 min after establishing pneumoperitoneum and the  steep Trendelenburg position  T3: 30 min after establishing pneumoperitoneum and the  steep Trendelenburg position  T4: at the end of surgery after the desufflation of  pneumoperitoneum in the supine position   1. Variables;   ONSD, mean arterial blood pressure, heart rate, tympanic body temperature, static pulmonary compliance, dynamic pulmonary compliance, end-tidal CO_2_ partial pressure, arterial CO_2_ partial pressure, arterial O_2_ partial pressure, hemoglobin concentration, and regional cerebral oxygen saturation using near infrared spectroscopy. |
| **Statistical analysis** | 1. Continuous variables are expressed as mean ± SD. 2. ZEEP and PEEP group data are compared using Student’s t-test or Mann-Whitney U test for continuous variables and chi-square test or Fisher’s exact test for categorical variables as appropriate. 3. Two-way repeated measures analysis of variance with Holm-Sidak test for multiple comparisons is used to evaluate the changes in the ONSD, hemodynamic variables, respiratory variables, and rSO_2_ at T0, T1, T2, T3, and T4. 4. A P value < 0.05 is considered statistically significant. |

**Ethics**

The present study is conducted by the research protocol approved by Asan Medical Center Institutional Review Board and according to the principles expressed in the Declaration of Helsinki. Authors must protect the private information of subjects participating in the present study.

**Backgrounds**

Robot-assisted laparoscopic prostatectomy requires a pneumoperitoneum and the steep Trendelenburg position to facilitate a surgical field. These specific conditions induce decreased pulmonary functional residual capacity and pulmonary compliance, which are likely to impose postoperative respiratory complications. The lung protective ventilation strategy during surgery, which consists of low tidal volume and positive end-expiratory pressure ventilation, has been known to improve postoperative respiratory outcomes in diverse surgical patients, including those undergoing laparoscopic surgery, who are at high risk for postoperative respiratory complications.

Lung protective ventilation strategy during laparoscopic surgery is promising in its potential to improve outcomes in regards to pulmonary function. However, the application of positive end-expiratory pressure as an important component of this strategy does not necessarily favor the function of other major organs. In particular, it has been proposed that positive end-expiratory pressure results in increased intracranial pressure by impeding cerebrospinal flow outflow and cerebral venous drainage. Furthermore, recent studies have reported that the sonographic optic nerve sheath diameter, which reflects increased intracranial pressure, increased during robot-assisted laparoscopic prostatectomy. Thus, the application of positive end-expiratory pressure may induce an additional increase in increased intracranial pressure during pneumoperitoneum and the steep Trendelenburg position in patients undergoing robot-assisted laparoscopic prostatectomy. However, no reports have evaluated the relationship between positive end-expiratory pressure and increased intracranial pressure during specific conditions such as pneumoperitoneum and the steep Trendelenburg position.

**Purpose of study**

The purpose of this study is to evaluate the effect of positive end-expiratory pressure as a lung protective ventilation strategy on the sonographic optic nerve sheath diameter as a surrogate for intracranial pressure during pneumoperitoneum and the steep Trendelenburg position in patients who are scheduled to undergo robot-assisted laparoscopic prostatectomy.

**Expected periods of research**

From the approval date of Institutional Review Board to June 30, 2016

**Subjects and Methods**

**Subjects**

**Inclusion criteria**

Patients who are scheduled to undergo robot-assisted laparoscopic prostatectomy

**Exclusion Criteria**

Patients who have cerebrovascular disease or history of cerebrovascular disease

Patients who refuse to participate

Patients who are younger than 20 years

**Statistical analysis**

1. Continuous variables are expressed as mean ± SD.
2. ZEEP and PEEP group data are compared using Student’s t-test or Mann-Whitney U test for continuous variables and chi-square test or Fisher’s exact test for categorical variables as appropriate.
3. Two-way repeated measures analysis of variance with Holm-Sidak test for multiple comparisons is used to evaluate the changes in the ONSD, hemodynamic variables, respiratory variables, and rSO_2_ at T0, T1, T2, T3, and T4.
4. A P value < 0.05 is considered statistically significant.

**Sample size calculation**

Our previous study shows an ONSD during pneumoperitoneum and the Trendelenburg position of 4.9 mm (SD: 0.4 mm). We assume a 10% increase in the ONSD in the PEEP group compared with that in the ZEEP group, with reference to a previous study of patients who received a 7-8 ml/kg tidal volume with 8 cmH_2_O PEEP. Power analysis suggests that a minimum sample size of 34 patients will be required to detect a 0.5 mm (about 10% of 4.9 mm) difference in the mean ONSD between the ZEEP and PEEP groups with a power of 80% at a P < 0.05 level of significance. Expecting a dropout rate of about 10%, we aim to include 38 patients.

**Methods**

**Study design**

Randomized controlled trial

**Protocols**

When hemodynamically stable conditions are reached, measurements are taken as follows: 10 min after anesthetic induction in the supine position before random allocation (T0), 5 min after applying ventilation strategies according to a random allocation (T1), 5 min after establishing pneumoperitoneum and the steep Trendelenburg position (T2), 30 min after establishing pneumoperitoneum and the steep Trendelenburg position (T3), and at the end of surgery after the desufflation of pneumoperitoneum in the supine position (T4).

At each predetermined time point, we measure the following variables; ONSD, mean arterial blood pressure, heart rate, tympanic body temperature, static pulmonary compliance, dynamic pulmonary compliance, end-tidal CO_2_ partial pressure, arterial CO_2_ partial pressure, arterial O_2_ partial pressure, hemoglobin concentration, and regional cerebral oxygen saturation using near infrared spectroscopy.

**Randomization and Blindness**

One investigator generates a computer-generated randomization codes, enrolls participants, and assigns participants to a computer-generated randomization codes kept in sequentially numbered opaque envelopes. After anesthetic induction, these envelops are opened by an investigator who controls ventilator setting, and patients randomly allocates to one of two groups; Zero end-expiratory pressure (ZEEP) group received mechanical ventilation with a tidal volume of 8 ml/kg of ideal body weight without PEEP, and PEEP group received mechanical ventilation with a tidal volume of 8 ml/kg of ideal body weight with 8 cmH_2_O PEEP. One investigator controls the ventilator setting according to a randomization code and other investigators who are blinded to the ventilator setting measure sonographic the ONSD.

**Data analysis**

All of the statistical analyses are performed using SigmaPlot software, version 12.5 (Systat Software Inc, San Jose, CA, USA).

**Strategy for the protection of patients’ medical information**

The access of patients’ information should be limited to authorized researchers. Moreover, authors have great concerns to protect private information of the patient during the entire research period.

**Reference**
